# A Hybrid Deep Transfer Learning of CNN-Based LR-PCA for Breast Lesion Diagnosis via Medical Breast Mammograms

**DOI:** 10.3390/s22134938

**Published:** 2022-06-30

**Authors:** Nagwan Abdel Samee, Amel A. Alhussan, Vidan Fathi Ghoneim, Ghada Atteia, Reem Alkanhel, Mugahed A. Al-antari, Yasser M. Kadah

**Affiliations:** 1 Department of Information Technology, College of Computer and Information Sciences, Princess Nourah bint Abdulrahman University, P.O. Box 84428, Riyadh 11671, Saudi Arabia; nmabdelsamee@pnu.edu.sa (N.A.S.); geatteiaallah@pnu.edu.sa (G.A.); rialkanhal@pnu.edu.sa (R.A.); 2 Department of Computer Sciences, College of Computer and Information Sciences, Princess Nourah bint Abdulrahman University, P.O. Box 84428, Riyadh 11671, Saudi Arabia; 3 Biomedical Engineering Department, Helwan University, Cairo 11795, Egypt; Vidanfathighoneim@h-eng.helwan.edu.eg; 4 Department of Artificial Intelligence, College of Software & Convergence Technology, Daeyang AI Center, Sejong University, Seoul 05006, Korea; en.mualshz@sejong.ac.kr; 5 Electrical and Computer Engineering Department, King Abdulaziz University, Jeddah 22254, Saudi Arabia; ykadah@kau.edu.sa; 6 Biomedical Engineering Department, Cairo University, Giza 12613, Egypt

**Keywords:** hybrid CNN-based LR-PCA, deep feature extraction and reduction, breast lesion classification, CAD system, breast cancer

## Abstract

One of the most promising research areas in the healthcare industry and the scientific community is focusing on the AI-based applications for real medical challenges such as the building of computer-aided diagnosis (CAD) systems for breast cancer. Transfer learning is one of the recent emerging AI-based techniques that allow rapid learning progress and improve medical imaging diagnosis performance. Although deep learning classification for breast cancer has been widely covered, certain obstacles still remain to investigate the independency among the extracted high-level deep features. This work tackles two challenges that still exist when designing effective CAD systems for breast lesion classification from mammograms. The first challenge is to enrich the input information of the deep learning models by generating pseudo-colored images instead of only using the input original grayscale images. To achieve this goal two different image preprocessing techniques are parallel used: contrast-limited adaptive histogram equalization (CLAHE) and Pixel-wise intensity adjustment. The original image is preserved in the first channel, while the other two channels receive the processed images, respectively. The generated three-channel pseudo-colored images are fed directly into the input layer of the backbone CNNs to generate more powerful high-level deep features. The second challenge is to overcome the multicollinearity problem that occurs among the high correlated deep features generated from deep learning models. A new hybrid processing technique based on Logistic Regression (LR) as well as Principal Components Analysis (PCA) is presented and called LR-PCA. Such a process helps to select the significant principal components (PCs) to further use them for the classification purpose. The proposed CAD system has been examined using two different public benchmark datasets which are INbreast and mini-MAIS. The proposed CAD system could achieve the highest performance accuracies of 98.60% and 98.80% using INbreast and mini-MAIS datasets, respectively. Such a CAD system seems to be useful and reliable for breast cancer diagnosis.

## 1. Introduction

According to the global cancer statistics in 2020, GLOBOCAN 2020 [1], an estimated 19.3 million new cancer cases have been diagnosed worldwide, with over 10.0 million cancer deaths in 2020. With an anticipated 2.3 million new cases, female breast cancer has surpassed lung cancer as the most diagnosed malignancy, followed by lung, colorectal, prostate, and stomach cancers. In Saudi Arabia, breast cancer is the most common type of cancer, and in 2018, the incidence and death age-standardized rates for Saudi women were 27.3 and 7.5 per 100,000, respectively [2]. The most efficient way to diagnose breast cancer is medical imaging examination. Digital mammography, magnetic resonance imaging (MRI), ultrasound, and infrared thermography are some of the imaging techniques utilized for diagnosis of breast cancer, while mammography imaging is the most important paradigm for its early detection [3]. The overall goal is to enable early breast cancer treatment, improve survival rates, and limit the need for severe treatments such as mastectomy [4,5]. On a mammography, dense breast tissue can appear white or light gray. This can make mammograms more difficult to read in younger women with denser breasts [6]. Many breast disorders have symptoms that are similar to cancer and require testing and, in some cases, a biopsy to diagnose. When a mammography detects something that appears to be cancer but turns out to be benign, it is called a false positive result. Based on the density of the breasts, radiologists may diagnose incorrectly with a percentage of 30% of breast cancers [7]. It is challenging for even experienced radiologists to interpret a huge number of screening mammography. Masses and microcalcifications are two types of cancer markers that can be detected by mammography technology. However, the mass detection is thought to be more difficult than microcalcification detection, not only because of the wide range of size and shape that masses can take on in a mammography, but also because masses often have poor image contrast [6,8]. Therefore, the CAD, has been developed to assist radiologists in analyzing the entire images and highlighting probable areas of concern that require further investigation. CAD can detect tumors that a radiologist may miss. It can be considered a second opinion automated tool that can assist the radiologists by automatically identifying the areas of abnormal contrast, suspicious regions [9].

Artificial intelligence, or AI, is a well-known technology that has recently been used in the development of effective CAD systems for the classification of lesions in mammograms. Numerous researchers have lately developed AI-based solutions for the automated detection and diagnosis of breast anomalies in mammography pictures. These solutions can be divided into two types: classical and deep learning models. Although the deep learning approach has achieved exceptional performance in the context of medical image analysis, several problems remain, as discussed in [10]. Over-fitting, the high complexity of deploying deep learning models, and the loss of some visual information owing to the preprocessing phase are some of these concerns. Overfitting is a key issue when applying deep learning models to medical data because of the small data size in comparison to the large number of network parameters [11,12,13]. As a result, the use of pretrained CNN models is recommended in this work to alleviate over-fitting and reduce the required high complexity in training deep learning models from scratch. However, the number of retrieved features from pretrained CNN is huge, which may cause significant problems throughout the data processing process, such as the multicollinearity problem. Multicollinearity occurs when input features of a dataset are strongly correlated. This affects regression and classification model effectiveness. Multicollinearity is reduced in this study by the utilization of feature reduction such as the Principal Components Analysis, PCA. The use of PCA is empowered in this study by using the Logistic Regression to pick the significant principal components returned from the PCA analysis [14,15].

To highlight our contribution, this study extends our prior work [16] in constructing an effective CAD system for the categorization of lesions in breast mammograms and contributes the following:

- A hybrid deep learning model of CNN-based LR-PCA has been designed for breast lesion classification purpose using the X-ray breast cancer images (i.e., mammograms).
- Generating more useful and powerful input information based on pseudo-colored images to enrich the input knowledge of deep learning models.
- A comprehensive study of transfer learning based on multiple convolutional networks has been conducted in order to extract the high-level deep features directly from the pre-trained backbone models.
- Resolving the multicollinearity problem that occurs among the derived high-level deep features from pre-trained by introducing a new method called LR-PCA. The optimized PCs are selected to perform the final classification purpose.
- A comprehensive evaluation process of the proposed CAD system is performed using two different public benchmark datasets: INbreast and mini-MAIS.

## 2. Related Work

Artificial intelligence, AI, is a well-known technology that has recently been employed in developing innovative solutions in the healthcare sector. These solutions can help patients in receiving an earlier diagnosis and precise treatments while also increasing the efficiency and effectiveness of healthcare services. The governance and regulation of AI services in healthcare is a major concern. The BSI (body substance isolation) and AAMI (Association for the Advancement of Medical Instrumentation) are non-profit standardization organizations that are investigating the role for standards in the deployment of innovative AI solutions within the healthcare sector [17]. Regulations protect the society by guaranteeing that the medical technology placed on the market poses an acceptable and fair level of risk. The quality of data inputs is one of the main issues that has been reported by the Medicines and Healthcare products Regulatory Agency, MHRA, in partnership with the AAMI. MHRA is an executive agency responsible for ensuring that medicines and medical devices work and are acceptably safe. They have recommended that the AI solutions will only be effective, and safe if the data models are trained on high quality data. Data samples need to contain enough variety to fulfill regulators, patients, and professionals and avoid unintentionally any error or bias in the AI outputs. In addition, the level of complexity is another concern. Eventually, the AI developer should understand and articulate the type of AI technique that is being developed, and its deployment environment.

The AI-based solutions for the automated detection and diagnosis of breast abnormalities in mammography images have recently been proposed by numerous researchers. These solutions can be classified into classical and deep learning models. Early CAD systems have been developed using the classical machine learning models such as the research conducted in [18,19,20,21,22,23,24,25,26,27,28,29,30]. The basic framework in these studies consists of three stages including Region of Interest (ROI) extraction, feature extraction and selection, and classification stage using well-known classifiers such as K Nearest Neighbor (KNN), Support Vector Machine (SVM), Neural Network (NN), and Ensemble classifiers such as the work conducted by Kadah et al. [18,21,24,25,31]. In [18], a CAD system that can help radiologists diagnose microcalcification patterns in digitized mammograms earlier and faster than traditional screening systems has been introduced. The proposed approach involves three steps including ROI extraction (32 × 32 pixels), feature selection using the wavelet decomposition, and classification of benign/malignant microcalcifications. Traditional classification methods were used: K-NN, SVM, NN, and fuzzy. MIAS mammographic databases were used to evaluate the suggested approach. Wavelet transform and fuzzy-neural algorithms have been proposed in [19]. These strategies are based on globally/locally processing of image/ROI. Classification of the normal/abnormal, and microcalcification masses has been accomplished on the MIAS benchmarking dataset. In [21], an enhancement in the classifier performance of the normal/microcalcification of breast tissue has been performed by the utilization of voting-based KNN and SVM classification approaches. A hybrid approach of SVM and Linear Discriminant Analysis (LDA) has been utilized in designing a CAD system for breast lesion classification in [22]. The genetic algorithm approach has been utilized for the classification of breast cancer in [23,32]. Lately, the deep learning techniques were used in most recent studies in the context of CAD system of breast cancer [33,34,35,36]. This is a natural consequence of the outstanding performance retrieved using the deep learning methods in image classification [37]. The most popular paradigm of the deep learning technology in the medical imaging is transfer learning which is based on using pretrained CNN such as Inception V3 [38], AlexNet [39], VGG19/VGG16 [40], ResNet50 [41], and GoogleNet [42]. The pretrained CNNs have been already trained on natural images such as ImageNet. The transfer learning approach has been exploited extensively in the classification of mammograms [43,44,45,46,47,48] to improve the performance of CNN architectures created from scratch. Transfer learning is utilized to enhance the performance of a machine learning model from one domain by transferring knowledge from a related domain [49]. The main advantage of transfer learning is the improvement of classification precision and rapid execution of the training phase [48]. Better improvement in the performance of transfer learning in the classification of mammograms has been introduced by utilizing the fusion of features extracted using different types of pretrained CNNs [50,51]. Another enhancement has been proposed in the study [52] which proposed a deep feature fusion framework using pretrained a CNN followed by the principal component analysis (PCA) as a feature reduction step and the SVM for the classification of breast cancer. The utilization of PCA as a dimensionality reduction for the features extracted using transfer learning has been proposed in [53] and yielded better performance than the individual use of the pretrained CNN. It is well known that PCA is the original and dominant dimension reduction tool and has been employed in a numerous study for the classification of mammograms using the traditional machine learning techniques.

With the current artificial NN and optimization skills, large-scale deep NN have been successfully developed, with a greater performance with deeper depth [54]. However, there are some concerns that should be investigated to assure the effectiveness of such solutions before being deployed in the healthcare sector. Some of the drawbacks of the AI technology have been surveyed in [55,56,57,58]. Additionally, the current challenges of deep learning technologies in the context of medical image analysis have been reviewed and defined in [10]. That article reviewed the basic concepts of deep learning and the most recent advances in medical images. They have defined the current problems of deep learning in the medical field and proposed a perspective of the primary research focus. In this study, we are investigating some of these raised issues. The first issue is the overfitting which is a major problem in applying deep learning models on medical data due to the very small data size relative to the huge number of network parameters. Second, most of network parameters come from fully connected layers after the convolutional part of the network. The third issue is that the number of classes in medical data sets is usually very small, and in many cases, binary classification problems are very common. Another issue is that all medical images are in grayscale while pretrained deep networks have color image input layers, repeating grayscale images for all three color channels is done to match input requirements, which does not seem efficient. The last issue that we have observed moreover in the current literature is that most of the aforementioned studies have employed the preprocessing of the medical images before they are used as input to deep learning networks. This does not seem to be rational given that such preprocessing aims to enhance images for human viewers and that deep networks should be able to learn the best preprocessing on its own from original images. Losing information in original images as a result of such preprocessing is likely and hence performance may be limited by the choice of such preprocessing.

## 3. Methodology

In this study, a hybrid deep learning CNN-based LR-PCA for breast lesion classification is proposed. Such a CAD system involves three main stages. First, three-channel pseudo-colored images are prepared based on the input grayscale images allowing deep learning models to extract more powerful deep features for better diagnosis performance. Second, backbone deep convolutional networks are used to extract the high-level deep features based on the transfer learning strategy avoiding the overfitting problem [59]. For deep feature extraction and selection, multiple deep learning models are employed such as AlexNet, VGG, and GoogleNet. Third, the LR-PCA model is proposed to select the significant principal components among the extracted features to tackle the problem of multicollinearity among the derived features. The dimensionality of the high-level deep features extracted from the top convolution layers is reduced using the PCA technique to obtain a reasonable feature space of low dimensionality while representing the variance in the input data. Then, Logistic Regression (LR) is used for selecting the significant principal components (PCs) extracted from the PCA.

**Figure 1:**
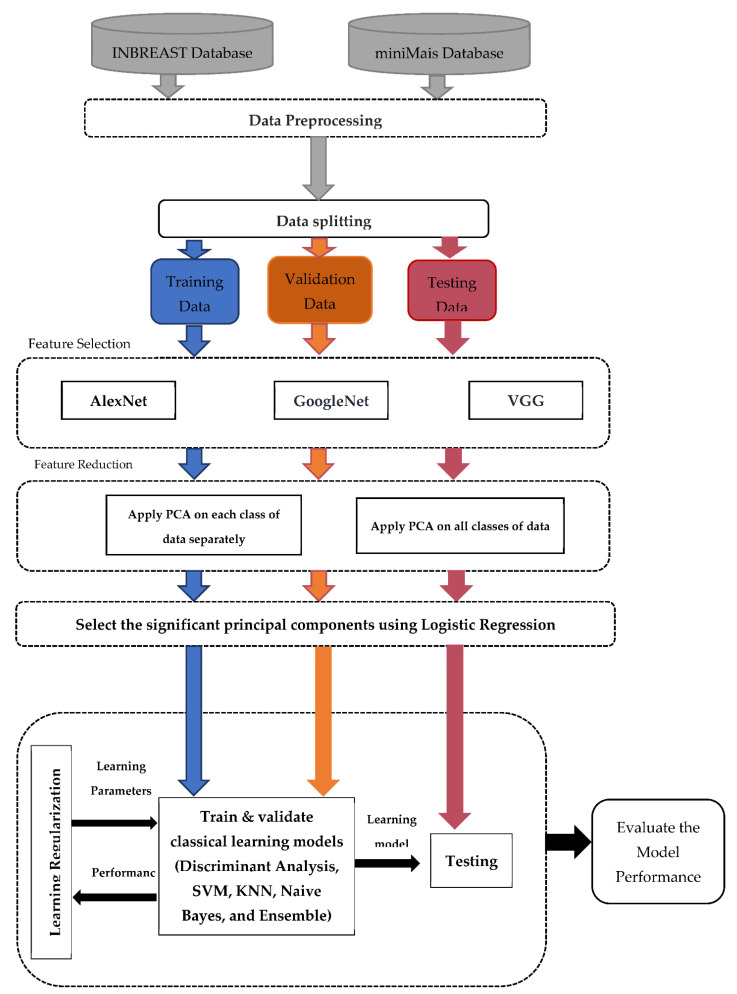
The Proposed CAD framework for breast lesion classification from X-ray mammograms.

The proposed CAD system comprises five modules including the preprocessing of images, features extraction using the pretrained CNN, dimensionality reduction using PCA, feature classification using the traditional machine learning techniques, and classifier evaluation using the performance metrics of CAD systems. The proposed framework is depicted in Figure 1.

### 3.1. Data Preparation

The data used in this work were obtained from different public datasets including the mini-MIAS for film-based, reduced resolution data, and the INbreast for full-field high-resolution digital mammography. The mini-MIAS is a popular Mammography Image Analysis Society (MIAS) database [60]. The mini-MIAS database was created from X-ray films carefully taken from the United Kingdom National Breast Screening Program. It was digitalized with to 50 microns resolution via a device having a linear optical density mapping of 3.2 and 8-bits per pixel quantization levels. However, the images were resized to 200-micron resolution and clipped/padded to retain a size of 1024 × 1024 pixels for all images. The database comprises right, and left breast images for 161 patients so the total number of images is 322. The images correspond to samples from normal, benign and malignant tissues with 208, 63 and 51 images correspondingly. The database is supplied with a ground truth diagnosis for all images and exact locations of abnormalities that may exist inside each image represented as the center and radius of the circle around each lesion. The mammograms that are included in the INbreast dataset were all obtained at a Breast Centre that is housed within a University Hospital [61]. It has a total of 410 mammograms, representing 115 distinct patient cases. Some of the cases were obtained from women who had both of their breasts examined, meaning that there were four mammograms performed on each woman. The INbreast includes a variety of lesions that can occur in the breast, such as masses, calcifications, and distortions of asymmetries. These lesions can be found in the breast. In addition to the type of lesion and the pathology type, the bounding box annotations of any existing lesions are present in each case.

The data preparation stage includes the following steps: Region of Interest, ROI, extraction, data augmentation, and generating pseudo-color images. ROIs fed to the CNN are derived from the suspicious areas of mammograms. Data augmentation is used to enlarge the number of samples in the training dataset. A 32 × 32 square region of interest (ROI) was extracted from the lesion. Such an ROI size was selected to directly compare our proposed framework against earlier work that addressed the same classification problem using various techniques [16]. However, the ROI selection method was commonly used for various medical image CAD system pipelines [62,63]. The selected ROI size could help in ensuring an adequate statistical representation of the breast lesion. The small ROI size makes it possible to subsequently allow better lesion localization capability for the developed models [32]. For the miniMIAS database, multiple ROIs were extracted and used from a single lesion whenever it was possible to obtain non-overlapping ROIs within the given lesion. This explains why the number of ROIs is larger than the number of images. We tried to avoid overlap to obtain completely independent ROIs and this was followed in previous work [16,62,63]. Regarding the INBreast database, the previous work on this database relied on extracting an ROI of the whole mass and resizing it to the input size of the deep learning model [64].

The created datasets contain 144 ROIs with equal number, 72 abnormal and normal regions for the mini-MIAS collection, and 34 and 73 benign/malignant ROIs for the INbreast dataset. Data augmentation, which was primarily based on the flipping (up/down and left/right) and rotation technique, was utilized in order to generate greater size of the dataset that were utilized. The ROIs have been rotated by multiple different angles: 0 degrees, 90 degrees, 180 degrees, and 270 degrees. The number of samples included in each category of the labeled ROI dataset that was generated is 576 and 1095 for the mini-MIAS and the INbreast, respectively.

**Figure 2:**
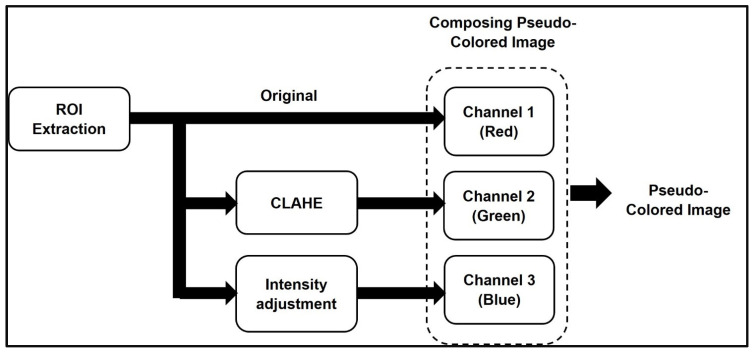
The concept of generating three-channel pseudo-color mapping image.

The pseudo-colored images were generated and used to obtain rich information allowing deep learning models to derive more accurate deep features. Instead of repeating the same image in all three-color channels, only one channel gets the original image while the other two channels receive different processed images that allow some additional global information to be incorporated within each pixel. The pseudo-color image display has been widely used in medical imaging since the digital imaging revolution and gives doctors new ways of detecting subtle variations because of the way colors are perceived by the human eye. Figure 2 illustrates the main steps that has been followed in generating the pseudo-color mapping which can be listed as follows:

- Channel 1 (Red) includes the original grayscale image.
- Channel 2 (Green) includes the enhanced image contrast of the grayscale image by transforming the values using contrast-limited adaptive histogram equalization (CLAHE) [65].
- Channel 3 (Blue) includes the processed image that maps the intensity values in the grayscale image to new values by saturating the bottom 1% and the top 1% of all pixel values. This operation increases the contrast of the output image.

**Figure 3:**
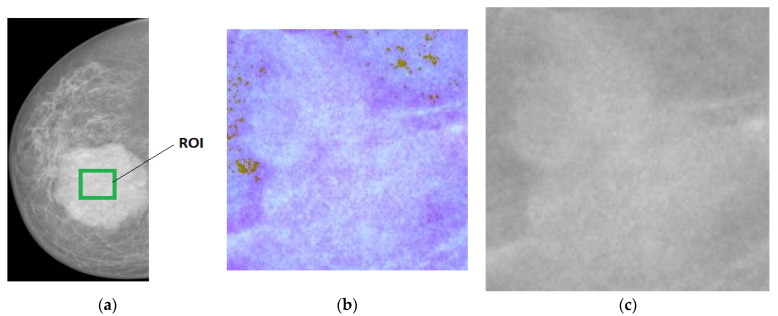
Example of data preparation phase for generating the pseudo-colored image based on the original grayscale X-ray mammogram. (**a**) Region of interest (ROI); (**b**) Pseudo-Colored image; (**c**) Grayscale image.

An example of an input image that was used during the data preparation phase can be seen in Figure 3, along with the corresponding pseudo-colored image that was generated as an output.

### 3.2. Feature Selection Using Transfer Learning

The conventional computer-aided diagnosing system in medical field relies heavily on the human designer’s efforts to extract handcrafted features, such as the density and shape of the cancer region [66]. This is a difficult task, as the extraction of handcrafted features is not significant in classifying the malignant areas despite the lengthy process [67]. Thus, other techniques such as transfer learning have been developed and utilized for selecting the significant features without the need for handcrafted features. The approach of transfer learning is based on utilizing pretrained CNNs in either the classification or extraction of the significant features in the training/validation dataset. There are three main types of layers in a pre-trained CNN: a convolutional layer, a pooling layer, and a fully connected (FC) layer. The convolution layers are used to extract the features, while a fully connected layer is used to classify them. This layer categorizes the selected features based on the input class. In this work, we have utilized the convolutional and pooling layers of pretrained CNNs in extracting the significant features in benign/malignant images of breast cancer; however, the FC layer has been replaced by traditional classifiers. AlexNet [39], VGG [40], and Googlenet [42] are some of the most popular pre-trained CNN models for image classification. Abdelhafiz et al. [68] have surveyed many research articles demonstrating their effectiveness in breast cancer classification. The AlexNet, VGG, and GoogleNet CNNs have been selected and utilized for this investigation due to their capability to generate high-level feature accuracy and relatively low processing complexity comparing with other models [68].

AlexNet [39] was introduced by Alex Krizhevsky and his team in 2012 in the ImageNet Large Scale Visual Recognition Challenge (ILSVRC) competition. Their CNN’s architecture is characterized by the fact that it has a much higher number of levels than previous models. There are five convolutional levels and three fully connected levels to this network’s input, which is an image with a resolution of 227 × 227 pixels. They have accomplished the top-5 error rates which is a significant improvement over what was previously possible.

VGG [40] has been introduced in the ImageNet Challenge 2014 by Karen Simonyan, and Andrew Zisserman. Using VGG, it has been shown that the depth of the network has a significant impact on CNN’s accuracy. The ReLU activation function has been inserted after the convolutional layers and followed by a pooling layer. Calibration is performed using softmaxes at the end of the model. VGG-11, VGG-16, and VGG-19 are three models of VGG-E with 11, 16, and 19 levels, respectively. The remaining 8, 13, and 16 levels in each model are convolution levels, with the final three levels of each model being fully connected.

GoogleNet [42] has also been introduced in ILSVRC challenge in 2014 by Szegedy C. et al. There are 22 layers in the GoogleNet CNN architecture. The Inception module serves as the foundation for this network structure, which is why it is known as Inception-v1. After nine inception units, each GoogleNet layer has a fully connected layer before it can be output. Inception modules are stacked on top of each other in GoogleNet, with a maximum layer of pooling. GoogleNet has twelve times fewer parameters than AlexNet, which makes it easier to train, even though it is more complex.

### 3.3. Dimensional Reduction Using LR-PCA

The number of extracted features from transfer learning when applied in the classification of medical images is huge. Although this creates opportunities to improve model performance, it also creates serious problems during the data analysis process. One of these problems is known as the multicollinearity problem [69]. Multicollinearity is a problem that arises when the input features of a dataset have a strong correlation with more than one of the other features in the dataset. The effectiveness of regression and classification models is hampered as a result of this factor. Feature reduction methods are the primary strategy that are implemented to reduce the effects of multicollinearity. PCA, or Principal Component Analysis, is a statistical technique that makes use of multicollinearity. In PCA, highly correlated variables are combined into a set of variables that are not correlated with one another. As a result, principal component analysis has the potential to successfully eliminate multicollinearity between features.

It is well known that principal component analysis is the original and preeminent dimension reduction technique, and that it has been utilized in numerous studies for the classification of mammograms utilizing conventional machine learning techniques. The process of PCA consists primarily of transforming the feature space through the relationship between attributes, mapping the initial feature space to the low-dimensional one to accomplish the goal of dimension reduction, and then analyzing the transformed feature space [70]. PCA is an unsupervised dimensional reduction approach, and it reduces the data dimension through the correlation between input features. The transformation matrix is optimized by finding the most significant differences in the original space.

The main principal in selecting the Principal Components, PCs, retrieved from the PCA is that the directions with the greatest variations contain the most information about classes. PCA is most commonly derived from a standard linear projection that maximizes the variance in the projected space. The most common method for selecting the PCs to be used is to first establish a threshold for the percentage of variance that can be explained, such as 80 percent, and then choose the number of components that generate a cumulative sum of variance that is as close as possible to that threshold. This is the standard approach to selecting the Principal Components to be used. This method suffers from the two primary weaknesses. It is necessary to make a personal decision regarding the threshold. The 80 percent or 90 percent thresholds are arbitrary and do not have a fair motive for being chosen in the majority of cases; instead, they are chosen at random. There is a chance that some portion of that variation is just noise and not an actual signal. It is not possible to know in advance whether or not the selected threshold eliminates only noise or it may remove important information from the signal itself. To overcome that problem, we are proposing a hybrid usage of Logistic Regression, LR, and PCA to select the significant components as a further reduction of features yielded from the transfer learning. LR is a common ML approach that can be utilized to explore the relationship between the input feature and outcomes. Logistic regression is a simple and effective binary classification model. It is very easy to implement LR and it works very well with classes that can be separated by a straight line. In this work, a Binomial Logistic Regression model has been constructed using a subset of the retrieved PCs from the PCA analysis. The selected PCs from the LR are the one that have a significant coefficient in the constructed LR model, *p*-value less than 0.05. As a result, the LR-PCA algorithm is as follows:

1. Create a covariance matrix for your dataset.
2. Determine the eigenvectors and eigenvalues of that matrix.
3. Choose the desired number of dimensions and filter the eigenvectors to match, sorting them by their associated eigenvalue.
4. Multiply the original space by the feature vector produced in the preceding step.
5. Build a Multinomial Logistic Regression model for the retrieved features.
6. Pick the principal components of the newly created LR model that have significant coefficients.

### 3.4. Classical ML Approaches for ROI Classification and Model Evaluation

In this work, the statistical machine learning system comprised the implementation of six distinct families of traditional classifiers. These classifiers included both parametric and nonparametric classification strategies. Methods such as decision trees, discriminant analysis, ensemble, KNN, naive Bayes, and SVM are examples of these types of techniques. By using 5-fold cross-validation to obtain a reliable estimation of the performance, the various parameters and variants that make up each classifier were optimized in order to achieve the best possible performance and reduce the issue of overfitting as much as possible. In each fold, different portions of images are assigned for each set. This process is carried out five times, which is the same as the number of folds, and the results obtained from each fold are averaged together to determine the overall performance of the system. In particular, the data for benign and malignant cases are randomly split into three sets including the training, validation, and testing sets. The percentage of division between the different splits are 70%, 15%, and 15% for training, validation, and testing sets, respectively.

To evaluate the proposed CAD framework, Accuracy (Acc.), sensitivity (SE), specificity (SP), False Negative Rate (FNR), False Positive Rate (FPR), Area Under the Curve (AUC), Matthews Correlation Coefficient (MCC), and F1-score were used [16,71]. All evaluation metrics were driven using the benefits of confusion matrix. Although accuracy is a significant performance metric due to the fact that it provides the percentage of correct classification in relation to the total number of samples, it does not distinguish between errors based on whether they are false-positive or false-negative. This presents a problem in the context of a CAD system, because in that scenario, a false-negative classification could have much more serious repercussions than a false-positive one. This issue is addressed by the sensitivity metric, which provides the percentage of malignant cases that were determined to be correctly diagnosed. However, at the other hand, the specificity reveals the proportion of typical patients whose conditions were accurately identified. When taken together, they present the full picture, which enables an observer to make direct comparisons between the various systems. In a CAD system, for instance, if two different systems have the same level of accuracy, the one that has a higher level of sensitivity is the one that is preferred.

## 4. Results and Discussion

In this study, we have proposed an innovative framework of a CAD system for the classification of breast cancer lesions by the use of a hybrid pretrained CNN and LR-PCA learning model. The framework comprises four stages including data preparation, feature extraction, feature reduction, and classification. In order to highlight the effect that the newly developed method in the data preparation stage has on the overall performance of the CAD system, we have generated two distinct sets of images for each database that was used in this investigation. These sets include grayscale and pseudo-colored images, respectively. In total, we have four different datasets being utilized in the testing of the adequacy of the introduced CAD system.

**Figure 4:**
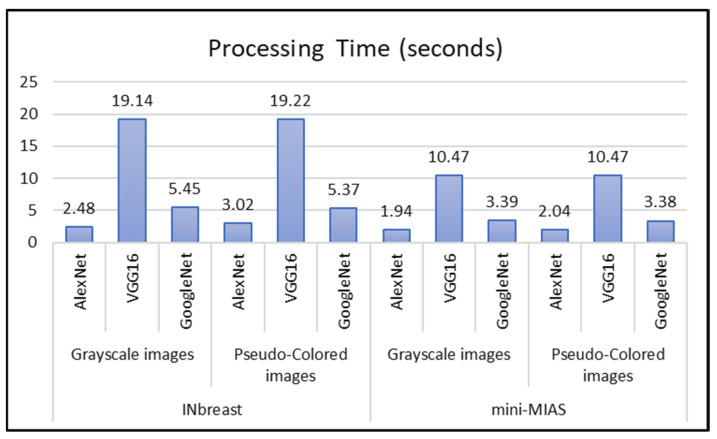
The retrieved processing time using Pretrained CNN for each dataset.

The pre-trained CNN has been employed as a feature extraction stage. The ROIs in each dataset have been resized to the corresponding size of each network (i.e., 227 × 227 for AlexNet and 224 × 224 for both VGG and GoogleNet) using bilinear interpolation with an anti-aliasing filter in trying to attain network requirements and preserve the image quality and maintain them free of aliasing artifacts, as the input layer must not be altered as a part of the transfer learning strategy. During the training of CNNs, the learning parameters should be estimated. In this work, a stochastic gradient descent with momentum, SGDM, optimizer was utilized. The learning rate for this optimizer was set to 0.0001, the momentum term factor was set to 0.9, the L2-Regularization setting was set to 0.0005, and the gradient threshold method used the L2-norm. The number of training epochs that could be performed was set at 100, and the mini-batch size that could be used was 16. These training options were chosen by examining the validation results obtained through the running experiments. They were applied across all networks to enable a direct comparison of the networks’ respective results as well as the training time incurred by each of them. Figure 4 outlines the required processing time for the feature extraction phase from each pre-trained deep learning model using both INbreast and mini-MIAS datasets. The development processing of this work has been executed using the academic version of Matlab 2020a coupled with the Machine Learning toolbox. The processor of the computer system is a quad-core Intel^®^ CoreTM i7-6700HQ that operates at 2.60 GHz. It also has 16 gigabytes of random access memory (RAM), and a CUDA-supported graphics card (NVIDIA GeForce GTX 950M with 4 gigabytes of memory). Although the retrieved processing time of the conducted experiments depends on the execution environment specifications, the insights gained could be reflected by comparing the derived overall performances of the alternative methodologies. As depicted in Figure 4, the VGG16 has consumed the largest processing time compared to the other networks. This is due to the fact that VGG16 possesses a network that is more complicated and deeper than that of the AexNet, and GoogleNet. In addition, we have observed that network architecture affects the quantity of features extracted. The number of retrieved features from AlexNet, GoogleNet, and VGG16 are 4096, 1024, and 4096 respectively. This is true regardless of the type of dataset that is being utilized.

**Figure 5:**
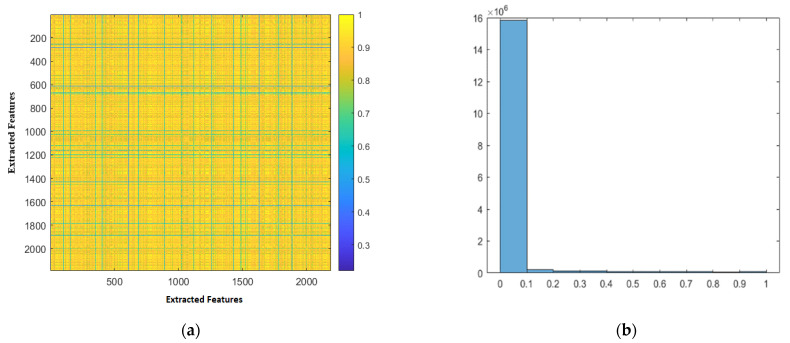
A heatmap for the correlation coefficient between the extracted features and a histogram of the corresponding *p*-value. (**a**) The heatmap of correlation coefficient; (**b**) Histogram of the *p*-value.

We have computed the correlation coefficients between the extracted features as well as the corresponding *p*-values for each feature pair in order to validate the necessity of applying principal component analysis and demonstrate that the extracted features are affected by the multicollinearity problem. The range of possible *p*-values is from 0 to 1, with values close to 0 indicating a significant correlation between the extracted features. For illustration, Figure 5 presents a heatmap depicting the retrieved correlation between the features extracted from one of the pretrained CNNs, as well as a histogram of the corresponding *p*-value. Figure 5 depicts the correlation coefficient using colors that are graded from lightest to darkest (yellow, and dark blue color represent the highest correlation coefficients). Most of the extracted features have high values for the correlation coefficient between their own and each other’s as represented by yellow color in the heatmap, as shown in the figure. Furthermore, the vast majority of the corresponding *p*-values for the correlation coefficient lie in the bin containing the numbers 0 to 0.1, which indicates that the coefficients are significant.

**Figure 6:**
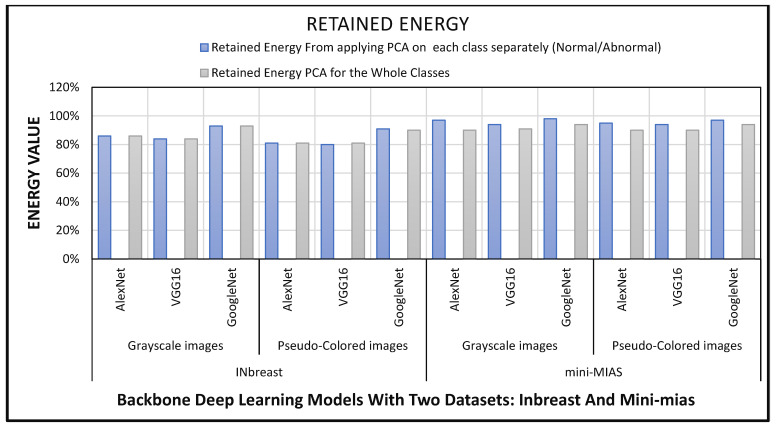
The energy retained within the retrieved PCs.

PCA is implemented by first computing the covariance matrix of the extracted features, and then calculating the PCs by employing eigenvalue decomposition. This is done so that PCA can more accurately analyze the data. After sorting the eigenvalues, the 50 values with the highest absolute value are chosen. The projection is carried out with the help of the corresponding eigenvectors. The 50 selected PCs has retained a high amount of energy within the components as illustrated in Figure 6. In order to achieve the best results from the PCA analysis, it is first performed on the training data from each class individually, and then it is combined with the principal components from all of the classes. As a result of this, the creation of class-specific features ought to be feasible at this point. It is obvious from Figure 6 that the retained energy within the PCs that is obtained by applying PCA to each category of cancer yields a higher amount of energy than the energy that is obtained by applying PCA to all of the classes combined.

**Figure 7:**
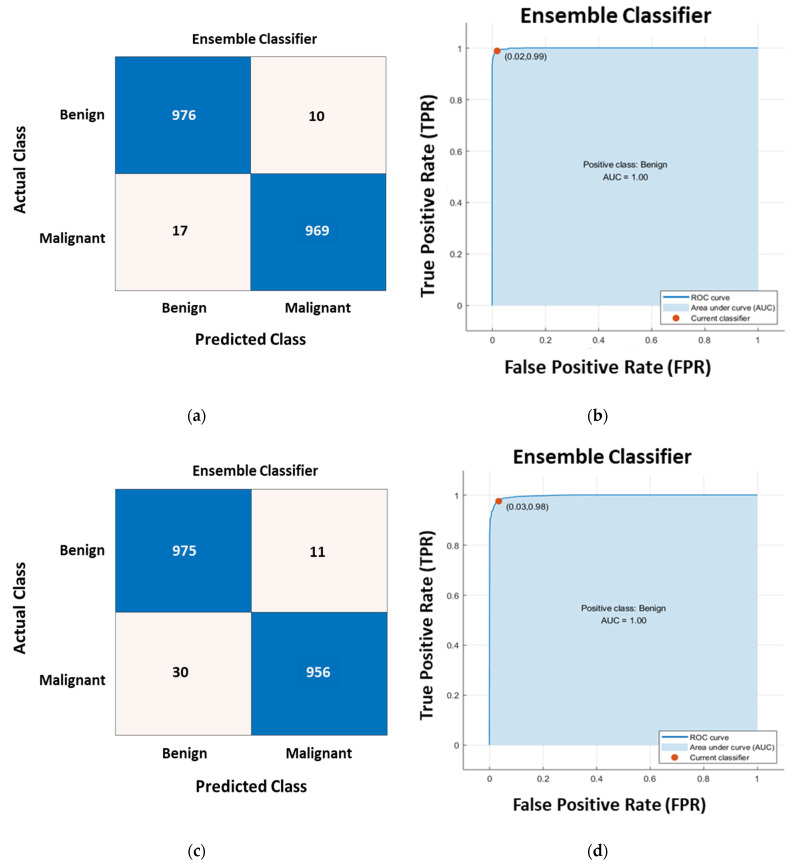
The confusion matrices and corresponding ROC curves of the classification results based on the proposed CAD system with deep extractor AlexNet and the LR-PCA. (**a**) The derived confusion matrix when the PCA is separately applied for each class; (**b**) The ROC curve when the PCA is separately applied for each class; (**c**) The confusion matrix when the PCA is applied across all classes; (**d**) The ROC curve when the PCA is applied across all classes.

Utilizing the Logistic Regression approach allowed for an additional level of filtering of the PCs to be carried out in an effective manner. Following the completion of the PCA analysis, a Binomial LR model was developed using the retrieved set of principal components (50 PCs). We have used the ANOVA F-statistics test as a method of hypothesis testing in order to evaluate the significance of the LR model in terms of choosing the significant PCs. This test was carried out so that we could assess the significance of the LR model. The significance level of the estimated coefficients that are yielded from the binomial logistic regression model has been measured. The significance has been calculated using the following metrics including the standard errors (SE), the t statistics (*t*), and the *p*-value of the coefficient estimates. The retrieved results of the test for the coefficient estimates of the fifty extracted principal components are presented in Table 1. The *p*-values that were retrieved show that the implanted LR model is effective in selecting the significant PCs that should be utilized in the subsequent classical classification model. The PCs that were chosen from the LR model were the ones that had a significant coefficient in the constructed LR model with a *p*-value that was lower than 0.05 as highlighted in gray color in Table 1. After that step, the total number of PCs that have been chosen is 23 which has been submitted to six distinct families of classical classifiers including the decision trees, discriminant analysis, SVM, KNN, naive Bayes, and Ensemble. We have performed three experiments in testing and evaluating the adequacy of the introduced CAD system. In the first experiment, the extracted features from the pretrained CNNs (AlexNet, VGG, and GoogleNet) are fed directly to the classical classifiers. In the second experiment, the extracted features are reduced using the LR-PCA before being submitted the classical classifiers and the PCA is applied for each class (normal/abnormal) separately. Additionally, in the last experiment, the PCA is utilized across all of the classes. The three experiments were carried out on a total of four image collections, which included the grayscale and pseudo-colored images derived from the mini-MIAS, and INbreast benchmarking datasets. Therefore, a total of twelve separate experiments were carried out in this work. The retrieved results yielded from experiments performed on the INbreast, and mini-MIAS datasets are presented in Table 2 and Table 3, respectively. The results that are presented in these tables are the ones that were retrieved from the best classification model out of the pool of classical classifiers that were implemented in this work. The utilization of LR-PCA on the extracted features using AlexNet for each class of the INbreast/mini-MAIS (pseudo-colored images) separately has yielded the best performance as highlighted in gray color in Table 2 and Table 3, respectively. The PCA has been applied in an optimized manner. The PCA is performed on training data from each class separately then principal components have been combined. This has allowed class-specific features to be developed and yielded those excellent results. For illustration, the ROC curve and confusion matrix for the classification results yielded from applying the LR-PCA on the extracted features from AlexNet are displayed in Figure 7.

## 5. Comparing the Performance and Conclusions

One of the real medical challenges of the AI applications is to build an automatic and accurate computer-aided framework for breast lesion diagnosis. In the body of published work, there have been great investigations and attempts to construct an accurate CAD system for such applications. The classification performance of the introduced system is further assessed in comparison to a number of state-of-the-art breast cancer detection systems as reported in Table 4. The early studies [72,73,74,75,76,77,78] focused mostly on identifying the textural features of breast tissues and applying traditional machine learning algorithms for classification purpose. The outcomes of those methods were not appropriated for the accurate classification of breast lesions since they lack high accuracy and sensitivity. However, the developed CAD system based on the classification of ROIs has yielded as high a performance as the work conducted using the classical ML methods [16,75] and the DL based methods [52,63,64,79,80,81,82,83]. For the MIAS dataset, the proposed LR-PCA-based system outperforms the deep learning systems developed by Ragab et al. [29], Alhussan et al. [16], and Zhang et al. [81]. Moreover, it has been noticed that the presented system achieved superior accuracy and sensitivity over the system developed by Zhang et al. [81] on the INbreast dataset. Generally, the comparison in Table 4 reveals that the proposed system could record a comparative performance with all other systems.

To conclude the current investigation, we have suggested a novel framework for a CAD system that can classify breast cancer lesions. This framework makes use of a hybrid pretrained CNN and LR-PCA learning model. The introduced system comprises four modules including data preparation, feature extraction, feature reduction, and classification. In the data preparation module, we have developed a new method for submitting the input images by applying the original ROI and the preprocessed ones in the input channels of the pretrained CNNs. The generated pseudo-colored images from the mini-MIAS and INbreast datasets, respectively, have been applied for the introduced CAD system and yielded better performance compared to the one achieved from the grayscale images. Three pretrained CNNs including the AexNet, GoogleNet, and VGG16 have been employed in the feature extraction phase and the number of extracted features from them is 4096, 1024, and 4096, respectively. These networks represent different architectures with different levels of complexity as represented by the number of parameters (weights and biases). In particular, the numbers of parameters for these networks are approximately 61 million for AlexNet, 7 million for GoogLeNet, and 138 million for VGG-16. Even with the least complex of them, the huge number of parameters suggests that it is not possible to properly train such networks with the limited data set available in this study and outline the value of using them as pretrained feature extractors rather than attempting to retrain them. Furthermore, these networks were used to classify the same data sets of this study through transfer learning. This allows direct comparison of the results. Naturally, there are additional options for pretrained CNNs that appear to be significant; nevertheless, it is challenging to address more models in a brief presentation.

A correlation analysis has been accomplished on the extracted features to examine the necessity for applying PCA on the feature matrix and demonstrating that the extracted features are affected by the multicollinearity problem. The Binomial Logistic Regression model has been developed using the retrieved set of principal components (50 PCs). The selected subset of these PCs is 23 according to their significance in the yielded results from the ANOVA F-statistics test on the developed LR model. The twenty-three PCs have been presented to six distinct families of traditional classifiers including the decision trees, discriminant analysis, SVM, KNN, naive Bayes, and Ensemble. Twelve classification experiments have been performed based on all features with or without the utilization of LR-PCA. The PCA has been examined with all classes as opposed to PCA with combined PCs that are class specific. The application of LR-PCA on the extracted features utilizing AlexNet for each class of the INbreast/mini-MAIS (pseudo-colored pictures) individually has provided the best performance. The performance of the proposed framework of our CAD system was compared to that of similar systems found in the literature, and the results of the comparison showed that the proposed one records the highest performance compared to all other systems.

## Figures and Tables

**Table 1:** Statistics of the Logistic Regression (LR) model.

Model Predictors	SE	*t* Statistics	*p*-Value
PC1	1.1638	7.8235	5.14 × 10^−^^15^
PC2	0.0008	14.7641	0
PC3	0.0013	−11.1861	4.77 × 10^−^^29^
PC4	0.0014	−13.4629	2.59 × 10^−^^41^
PC5	0.0019	−12.9322	2.96 × 10^−^^38^
PC6	0.0015	−2.1592	0.030837
PC7	0.0022	13.8375	1.51 × 10^−^^43^
PC8	0.0019	−0.0055	0.995623
PC9	0.0023	−9.0287	1.74 × 10^−^^19^
PC10	0.0026	1.2018	0.22944
PC11	0.0026	−2.1862	0.028801
PC12	0.0027	1.6188	0.1055
PC13	0.0032	−7.2077	5.69 × 10^−^^13^
PC14	0.0033	−8.3964	4.61 × 10^−^^17^
PC15	0.0034	−0.7858	0.431973
PC16	0.0035	1.8389	0.065923
PC17	0.0037	0.0854	0.931964
PC18	0.0036	5.9441	2.78 × 10^−^^9^
PC19	0.0035	1.8050	0.07108
PC20	0.0036	−6.9300	4.21 × 10^−^^12^
PC21	0.0040	4.9057	9.31 × 10^−^^7^
PC22	0.0040	−4.4528	8.47 × 10^−^^6^
PC23	0.0043	11.6463	2.40 × 10^−^^31^
PC24	0.0045	−4.8968	9.74 × 10^−^^7^
PC25	0.0048	−9.4881	2.35 × 10^−^^21^
PC26	0.0047	0.7119	0.476501
PC27	0.0047	−0.6445	0.519277
PC28	0.0050	−2.6172	0.008865
PC29	0.0053	0.9396	0.347413
PC30	0.0054	−6.5354	6.34 × 10^−^^11^
PC31	0.0050	−1.4328	0.151915
PC32	0.0054	−0.5211	0.602287
PC33	0.0052	2.2693	0.023249
PC34	0.0057	−0.6697	0.503072
PC35	0.0058	−0.1642	0.869595
PC36	0.0056	6.0392	1.55 × 10^−^^9^
PC37	0.0059	−1.5046	0.132437
PC38	0.0061	−2.5012	0.012377
PC39	0.0060	0.5841	0.559178
PC40	0.0062	2.9399	0.003284
PC41	0.0065	3.0751	0.002104
PC42	0.0065	−6.5521	5.67 × 10^−^^11^
PC43	0.0065	−3.3909	0.000697
PC44	0.0068	−0.2496	0.802873
PC45	0.0063	3.0721	0.002125
PC46	0.0065	−1.5325	0.125403
PC47	0.0064	−1.5169	0.1293
PC48	0.0067	−2.3565	0.01845
PC49	0.0069	1.7933	0.072918
PC50	0.0071	−3.3454	0.000822

**Table 2:** Classification evaluation performance (%) of the proposed CAD system with three different backbone deep learning classifiers. These results were derived using INbreast dataset.

Dataset	LR-PCA	Feature Extractor	Classification Model	Acc.	SE	SP	PRE	FNR	FPR	AUC	MCC	F1-Score
**Grayscale images**	Across all classes	AlexNet	Ensemble (subspace KNN)	97.20	96.75	97.67	97.65	3.25	2.33	100	94.43	97.20
		VGG16	Ensemble (subspace KNN)	95.90	94.12	97.77	97.68	5.88	2.23	99.0	91.95	95.87
		GoogleNet	Ensemble (subspace KNN)	93.90	92.29	95.54	95.39	7.71	4.46	98.0	87.88	93.81
**Pseudo-Colored images**		AlexNet	Ensemble (subspace KNN)	98.00	96.96	98.88	98.86	3.04	1.12	95.22	95.86	97.90
		VGG16	Ensemble (subspace KNN)	97.90	96.65	99.09	99.06	3.35	0.91	100	95.77	97.84
		GoogleNet	Ensemble (subspace KNN)	95.10	92.90	97.06	96.93	7.10	2.94	99.0	90.04	94.87
**Grayscale images**	For each class separately	AlexNet	Ensemble (subspace KNN)	97.20	96.65	97.67	97.64	3.35	2.33	100	94.33	97.15
		VGG16	Ensemble (subspace KNN)	95.90	95.13	96.65	96.60	4.87	3.35	99.0	91.80	95.86
		GoogleNet	Ensemble (subspace KNN)	94.60	92.90	97.06	96.93	7.10	2.94	99.0	90.04	94.87
**Pseudo-Colored images**		AlexNet	Ensemble (subspace KNN)	98.60	98.28	98.99	98.98	1.72	1.01	100	97.26	98.63
		VGG16	Ensemble (subspace KNN)	98.10	97.87	98.28	98.27	2.13	1.72	99.0	96.15	98.07
		GoogleNet	Ensemble (subspace KNN)	94.50	92.60	96.45	96.31	7.40	3.55	98.0	89.11	94.42
**Grayscale images**	Not applied	AlexNet	KNN	96.00	94.61	97.67	97.59	5.39	2.33	96.0	92.33	96.07
		VGG16	KNN	95.80	94.53	97.16	97.09	5.47	2.84	99.0	91.72	95.79
		GoogleNet	KNN	93.40	92.01	95.02	94.89	7.99	4.98	98.0	87.06	93.43
**Pseudo-Colored**		AlexNet	KNN	96.80	95.13	98.48	98.43	4.87	1.52	96.0	93.66	96.75
		VGG16	KNN	96.40	95.13	97.67	97.61	4.87	2.33	96.0	92.83	96.35
		GoogleNet	KNN	93.90	92.49	95.23	95.10	7.51	4.77	94.0	87.76	93.78

**Table 3:** Classification evaluation performance (%) of the proposed CAD system with three different backbone deep learning classifiers. These results were derived using mini-MIAS dataset.

Dataset	LR-PCA	Feature Extractor	Classification Model	Acc.	SE	SP	PRE	FNR	FPR	AUC	MCC	F1-Score
**Grayscale images**	Across all classes	AlexNet	Ensemble (subspace KNN)	97.50	99.42	96.38	96.41	0.58	3.62	100	95.81	97.89
		VGG16	Ensemble (subspace KNN)	97.30	97.49	97.30	97.30	2.51	2.70	100	94.79	97.40
		GoogleNet	Ensemble (subspace KNN)	97.50	97.47	95.98	95.98	2.53	4.02	100	93.45	96.72
**Pseudo-Colored images**		AlexNet	Ensemble (subspace KNN)	98.60	99.41	97.49	97.54	0.39	2.51	100	97.13	98.57
		VGG16	Ensemble (subspace KNN)	98.20	98.26	98.07	98.07	1.74	1.93	100	96.33	98.17
		GoogleNet	Ensemble (subspace KNN)	97.50	97.88	97.10	97.13	2.12	2.90	100	94.98	97.50
**Grayscale images**	For each class separately	AlexNet	Ensemble (subspace KNN)	98.10	98.65	97.49	97.51	1.35	2.51	100	96.14	98.08
		VGG16	Ensemble (subspace KNN)	97.00	98.07	96.53	96.58	1.93	3.47	100	94.61	97.32
		GoogleNet	Ensemble (subspace KNN)	96.30	96.35	96.71	96.72	3.65	3.29	99.0	93.05	96.53
**Pseudo-Colored images**		AlexNet	Ensemble (subspace KNN)	98.80	99.62	98.26	98.28	0.58	1.74	100	97.69	98.85
		VGG16	Ensemble (subspace KNN)	97.70	98.46	96.91	96.96	1.54	3.09	99.0	95.38	97.70
		GoogleNet	Ensemble (subspace KNN)	97.30	97.30	97.30	97.30	2.70	2.70	0.99	94.59	97.30
**Grayscale images**	Not applied	AlexNet	KNN	97.00	98.65	96.91	96.97	1.35	3.09	96.0	95.57	97.80
		VGG16	KNN	97.80	97.88	97.68	97.69	2.12	2.32	100	95.56	97.78
		GoogleNet	KNN	97.80	97.68	97.88	97.87	2.32	2.12	98.0	95.56	97.78
**Pseudo-Colored**		AlexNet	KNN	97.20	97.90	96.93	96.97	2.10	3.07	95.0	94.83	97.43
		VGG16	KNN	97.60	97.87	97.88	97.87	2.13	2.12	100	0.9575	0.9787
		GoogleNet	KNN	97.90	97.67	97.50	97.49	2.33	2.50	99.0	0.9517	0.9758

**Table 4:** Evaluation comparison results of the proposed CAD system against the state-of the-art breast cancer classification systems.

Reference	Feature Extraction Approach	Classifier	Dataset	SE (%)	Acc. (%)
Oliver et al. [78]	Utilizing Fuzzy C Means for lesion segmentation and a number of textural and morphological features are used	SVM	MIAS	87.33	91.51
Phadke et al. [77]	Breast cancer classification based on the fusion of local and global morphological and textural features	SVM	MIAS	92.71	83.1
Jian et al. [75]	Utilizing the wavelet transform in order to retrieve the textural features of ROIs	Classification of ROIs using SVM	MIAS	96.3	97.7
Vijayarajeswari et al. [76]	Breast lesions were classified using SVM after applying the Hough transform for feature extraction.	SVM	MIAS	-	94.0
Xie et al. [74]	Classification of breast lesions using metaheuristic-based classifier	PSO-SVM	MIAS	92.0	89.0
Mina et al. [73]	Classification of breast cancer using ANN and wavelet decomposition for feature extraction	ANN	MIAS	68.0	-
Liu et al. [72]	Detection of microcalcification in digital mammograms	SVM	INbreast	92.0	-
Xu et al. [79]	Deep CNN for feature extraction and classification of breast lesions	CNN	INbreast	-	96.8
Al-antari et al. [63,82]	End-to-end CAD system for the segmentation and classification of breast masses	YOLO classifier	INbreast	95.64	89.91
Ragab et al. [29]	Deep features fusion of AlexNet, GoogleNet, ResNet-18, ResNet-50, and ResNet-10.	SVM	CBIS-DDSM MIAS	98.0 99.0	97.90 97.40
Alhussan et al. [16]	AlexNet	AlexNet	MIAS	98.26	98.26
	GoogLeNet	GoogLeNet		98.26	98.26
	VGG-16	VGG-16		98.70	98.28
Zhang et al. [81]	Features were extracted by Gist, SIFT, HOG, LBP, VGG, ResNet, and DenseNet and fused together.	SVM, XGBoost, Naïve Bayes, k-NN, DT, AdaBoosting	CBIS-DDSM INbreast	98.61 57.2	90.91 87.93
Song et al. [83]	GoogleNet Inception-v2	XGBoost	DDSM	99.74	92.8
Khan et al. [80]	Fusion of deep features extracted by VGG-16, VGG-19, GoogleNet, and ResNet-50.	Transfer Learning	CBIS-DDSM MIAS	98.07	96.6
**Proposed CAD system**	**Features are selected using LR-PCA from pseudo images.**	**Hybrid Transfer Learning of CNN-based LR-PCA**	**MIAS INbreast**	**99.62 98.28**	**98.80** **98.62**

## Data Availability

Not applicable.
